# RNA-sequencing analysis revealed genes associated drought stress responses of different durations in hexaploid sweet potato

**DOI:** 10.1038/s41598-020-69232-3

**Published:** 2020-07-28

**Authors:** Mohamed Hamed Arisha, Muhammad Qadir Ahmad, Wei Tang, Yaju Liu, Hui Yan, Meng Kou, Xin Wang, Yungang Zhang, Qiang Li

**Affiliations:** 1 Xuzhou Institute of Agricultural Sciences in Jiangsu Xuhuai District/Key Laboratory of Biology and Genetic Breeding of Sweetpotato/Ministry of Agriculture and Rural Affairs/Sweetpotato Research Institute, CAAS, Xuzhou, 221131 Jiangsu China; 2 Department of Horticulture, Faculty of Agriculture, Zagazig University, Sharkia, 44511 Egypt; 3 Department of Plant Breeding and Genetics, Bahauddin Zakariya University, Multan, 60000 Pakistan

**Keywords:** Biotechnology, Genetics, Plant sciences

## Abstract

Purple-fleshed sweet potato (PFSP) is an important food crop, as it is a rich source of nutrients and anthocyanin pigments. Drought has become a major threat to sustainable sweetpotato production, resulting in huge yield losses. Therefore, the present study was conducted to identify drought stress-responsive genes using next-generation (NGS) and third-generation sequencing (TGS) techniques. Five cDNA libraries were constructed from seedling leaf segments treated with a 30% solution of polyethylene glycol (PEG-6000) for 0, 1, 6, 12, and 48 h for second-generation sequencing. Leaf samples taken from upper third of sweet potato seedlings after 1, 6, 12, and 48 h of drought stress were used for the construction of cDNA libraries for third-generation sequencing; however, leaf samples from untreated plants were collected as controls. A total of 184,259,679 clean reads were obtained using *second and third-generation* sequencing and then assembled into 17,508 unigenes with an average length of 1,783 base pairs. Out of 17,508 unigenes, 642 (3.6%) unigenes failed to hit any homologs in any databases, which might be considered novel genes. A total of 2, 920, 1578, and 2,418 up-regulated unigenes and 3,834, 2,131, and 3,337 down-regulated unigenes from 1 h, 6 h, 12 h, and 48 h library were identified, respectively in drought stress versus control. In addition, after 6, 12, and 48 h of drought stress, 540 up-regulated unigenes, 486 down-regulated unigenes and 414 significantly differentially expressed unigenes were detected. It was found that several gene families including Basic Helix-loop-helix (bHLH), basic leucine zipper (bZIP), Cystein2/Histidine2 (C2H2), C3H, Ethylene-responsive transcription factor (ERF), Homo domain-leucine zipper (HD-ZIP), MYB, NAC (NAM, ATAF1/2, and CUC2), Thiol specific antioxidant and WRKY showed responses to drought stress. In total, 17,472 simple sequence repeats and 510,617 single nucleotide polymorphisms were identified based on transcriptome sequencing of the PFSP. About 96.55% of the obtained sequences are not available online in sweet potato genomics resources. Therefore, it will enrich annotated sweet potato gene sequences and enhance understanding of the mechanisms of drought tolerance through genetic manipulation. Moreover, it represents a sequence resource for genetic and genomic studies of sweet potato.

## Introduction

Purple-fleshed sweet potato (PFSP) (*Ipomoea batatas* (L.) Lam*.*) is a type of sweet potato, which has purple skin and flesh color of the storage roots due to the large accumulation of anthocyanin pigments^1^. It is a root crop widely grown in some Asian and African countries (i.e. China, India, and Kenya). Sweet potato is regarded as the seventh most important food crop. It is among the crops which generate large amounts of food per unit area per unit time^2^. Furthermore, it is considered to be a cheap source of energy, carbohydrates, vitamin, potassium, iron, fiber, and protein^3^. Purple-fleshed sweet potato (PFSP) is also an important source of anthocyanin with strong antioxidant properties. Therefore, it can be a quick solution for nutritional problems in many developing countries^4^. Due to high anthocyanin content, sweet potato shows excellent antioxidant properties.

Fluctuations in climatic conditions and unprecedented rainfall have resulted in increases in the exposure of crop plants to different types of abiotic stresses, such as drought, salt, high temperature, and cold stress. Among these abiotic stresses, drought is the major yield-limiting factor in a variety of crop plants, including sweet potato. Drought stress causes plant water deficits in sweet potato, which results in decreased cell turgor and cell enlargement, closed stomata, decreased photosynthetic rate, and shortened vegetative period^5^. Moreover, it adversely affects anatomical traits of plants, physiological processes, biochemical pathways and ultimately inhibits plant growth and incurs severe yield losses^6^. Drought tolerance in sweet potato remains a challenge for plant breeders due to its large homogeneity of the chromosome number (2n = 6X = 90) with the estimated genome size of more than 2.4 GB. Furthermore, it expresses self-incompatibility with greater levels of cross-incompatibility^7^.

In non-model plants without a reference genome such as sweet potato, transcriptome sequencing approaches adopt a rapid method to improve drought tolerance through genetic manipulation^8^. Transcriptome sequencing is considered an effective strategy for studying the expression of a large number of genes under drought stress conditions^9^. Furthermore, RNA sequencing using high throughput next-generation and third-generation sequencing technologies can be used for the discovery of genes involved in drought tolerance and their related signaling pathways^10^. Transcriptome studies of a few cultivars of sweet potato have been conducted by de novo transcriptome assembly using second and third-generation sequencing technologies with Illumina platforms^11–18^. However, transcriptome sequencing was mostly performed from Ipomoea trifida (2n = 2x = 30), the closest wild relative of Ipomoea batatas, thus making it the sweet potato progenitor^19^.

In the present study, second and third-generation sequencing technologies were used to establish a useful database for transcriptome sequencing and differentially expressed genes in purple-fleshed sweet potato leaves under different durations of drought stress. A total of 184,259,679 high-quality reads were assembled into 18,209 transcripts and 17,508 unigenes. Our results provide novel insights into sweet potato response dynamics of changes to drought stress and revealed potential defense mechanisms of specific genes involved in drought tolerance under different time points. Their role in drought tolerance can help guide future efforts to breed for drought-tolerance in sweet potato.

## Materials and methods

### Plant materials and growth conditions

In this study, Xuzi-8, a high yielding drought tolerant and early maturing sweet potato cultivar with storage roots having purple flesh was used. Its storage roots contain about 6% soluble sugar content and more than 80 mg anthocyanin content/100 g fresh weight. This cultivar was obtained from Xuzhou Institute of Agricultural Sciences in Jiangsu Xuhuai District, China.

This sweet potato cultivar was propagated by apical stem cuttings of 15–20 cm length. The cuttings were grown hydroponically in the Hoagland nutrient medium for four weeks. The cultures were incubated at 25 ± 3 °C under 16 h light/8 h dark photoperiod. Seedlings were then exposed to drought stress by the addition of 30% PEG-6000 to Hoagland solution. Leaf samples (0.1 g) were collected from the upper third of the plant at 0 (control), 1, 6, 12 and 48 h after drought treatment and were used for next-generation sequencing. However, leaf samples taken from the upper third of sweet potato seedlings after 1, 6, 12 and 48 h of drought exposure were mixed and used for third-generation sequencing; untreated control samples were collected and examined in parallel. All samples were frozen in liquid nitrogen immediately upon collection and stored at − 80 °C until processing for RNA extraction. Each sample was analyzed in triplicate.

### Total RNA isolation

Total RNA was extracted from both drought treated and control plant leaves using the TRIZOL reagent (Life Technologies, Carlsbad, CA) according to the manufacturer’s protocol. RNA degradation and contamination were monitored on 1% agarose gels. RNA purity was checked using the Nanophotometer spectrophotometer (IMPLEN, CA, USA). The Qubit RNA Assay Kits were used with the Qubit 2.0 Fluorometer (Life Technologies, CA, USA) to determine RNA concentration. The RNA integrity was assessed using the RNA 6,000 Nano Kit of the Agilent 2,100 Bio-analyzer system (Agilent Technologies, CA, USA).

### cDNA library preparation for transcriptome analysis

For second generation sequencing, a total amount of 3 µg RNA per sample was used as input material for the RNA sample preparations. NEBNext Ultra RNA Library Prep Kit for Illumina (NEB, USA) was used for generating sequencing libraries following the manufacturer’s protocol and index codes were added to attribute sequences to each sample^20^. For third generation sequencing RNA was extracted from different time points and equal amount of each sample were mixed to produce one library beside to the control library. Briefly, poly-T oligo-attached magnetic beads was used to purify mRNA from total RNA. NEBNext First Strand Synthesis Reaction Buffer (5X) under elevated temperature was used for fragmentation through divalent cations. First-strand cDNA was synthesized using random hexamer primer and M-MuLV Reverse Transcriptase (RNase H-)^21^. Second strand cDNA synthesis was subsequently performed using DNA Polymerase I and RNase H. Remaining overhangs were converted into blunt ends via exonuclease/polymerase activities. After that, NEBNext Adaptor with a hairpin loop structure was ligated to prepare for hybridization. The library fragments were purified with the AMPure XP system (Beckman Coulter, Beverly, USA) to selected the fragments more than 250–200 bp. After that, 3 µL USER Enzyme (NEB, USA) was used with size-selected, adaptor-ligated cDNA at 37 °C for 15 min followed by 5 min at 95 °C before PCR. Universal PCR primers and Index (X) Primer. Finally, PCR products were purified and library quality was assessed on the Agilent Bioanalyzer 2,100 system.

### Sequence clustering and analysis

The clustering of the index-coded samples was performed on a cBot Cluster Generation System using TruSeq PE Cluster Kit v3-cBot-H (Illumina) following the manufacturer’s instructions. After cluster generation, the library preparations were sequenced on an Illumina platform and paired-end reads were generated.

### Quality control data analysis

At first, raw data (raw reads) of fastq format were processed through in-house Perl scripts. In this step, reads containing adapter, ploy-N, and low-quality reads were removed from raw data to obtain clean data (clean reads)^22^. The Q20, Q30, GC-content and sequence duplication level of the clean data were simultaneously calculated. All downstream analyses were based on clean, high-quality data^23^.

### Transcriptome assembly and gene functional annotation

Transcriptome sequencing was accomplished based on both NGS and 3rd GS, and TPM, FPKM, RPKM and fold change (FC) were recorded for each replicate of each library separately. Obtained sequence from NGS and 3rd GS was aligned and similar sequence data from all libraries/samples were pooled^24^ and ultimately used for further analysis using Trinity^25^ with min_kmer_cov set to 2 by default and all other parameters set default.

The functional annotation of genes was performed based on different source databases such as NCBI non-redundant protein sequences (Nr) (https://www.ncbi.nlm.nih.gov/) with an e-value cut-off of 1E-5; Nt (NCBI non-redundant nucleotide sequences) (https://www.ncbi.nlm.nih.gov/) with an e-value cut-off of 1E-5; Pfam (Protein families), (https://pfam.sanger.ac.uk/) with an e-value cut-off of 1E–2; KOG/COG (Clusters of Orthologous Groups of proteins) (https://www.ncbi.nlm.nih.gov/cog/) with an e-value cut-off of 1E–3 and Swiss-Prot (reviewed) (A manually annotated and non-redundant protein sequence database) (https://www.ebi.ac.uk/uniprot/) with an e-value cut-off of 1E–5.

Based on the annotation result of NR and Pfam, Blast2GO (v2.5) was used to determine GO (Gene Ontology) annotations (https://www.geneontlology.org) according to the three GO ontologies (molecular function, biological process, and cellular component)^26^. GO enrichment analysis of the differentially expressed genes (DEGs) was performed by the GO-seq R packages based on Wallenius non-central hypergeometric distribution^27^, which can adjust to gene length bias in DEGs.

Kyoto Encyclopedia of Genes and Genomes (KEGG)^28^ is a database resource for understanding high-level functions and utilities of the biological system, such as the cell, the organism and the ecosystem, from molecular-level information, especially large-scale molecular datasets generated by genome sequencing and other high-throughput experimental technologies (https://www.genome.jp/kegg/). The KOBAS software was used^29^ to test the statistical enrichment of DEGs in KEGG pathways.

### Quantification of gene expression levels and differential expression analysis

Gene expression levels were estimated using RSEM^30^ for each sample. Clean data were mapped back onto the assembled transcriptome. Read counts for each gene were obtained from the mapping results.

Differential expression analysis of two conditions/groups was performed using the DESeq2R differential expression package for determining differential expression in digital gene expression data using a model based on the negative binomial distribution. The obtained *P* values were adjusted using the Benjamini and Hochberg method for controlling the false discovery rate (FDR). Genes with the FDR-adjusted *P* value of ≤ 0.05 were defined as differentially expressed genes (DEGs)^31^. FDR ≤ 0.05 and log_2_ of the fold change (|log_2_ FC|) ≥ 1 were set as the thresholds for significant differential expression.

## Results

### Transcriptome sequencing, assembly, and mapping

**Figure 1:**
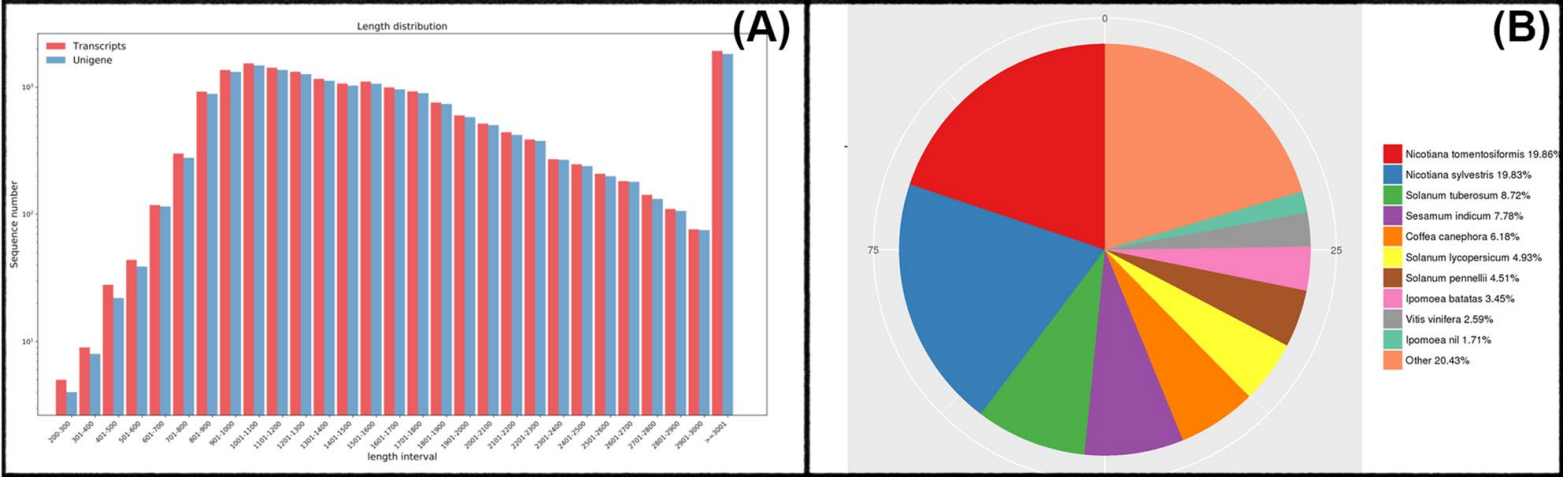
(**A**) Assembly result sequence length distribution map of transcripts and unigenes in Xuzi-8 sweetpotato cultivar under drought stress conditions. The horizontal axis represents the length intervals of the transcripts and unigenes, and the vertical axis represents the number of transcripts and unigenes. (**B**) Species distribution of the top BlastX matches of the transcriptome unigenes in the non-redundant protein database (Nr) data base under drought stress conditions.

In order to conduct gene expression profiling of hexaploid sweetpotato exposed to PEG-induced drought stress at different time points, ten cDNA libraries were constructed for NGS from leaves of treated (with 30% PEG-6000) and untreated seedlings after 0, 1, 6, 12, and 48 h. For 3rd GS, four time points RNA samples including1, 6, 12 and 48 h were mixed to produce one library beside to the control library. These libraries were separately sequenced using Illumina high-throughput third generation sequencing platform. TPM, FPKM, RPKM and fold change (FC) were recorded for each replicate of each library separately on both NGS and 3rd GS. Obtained sequence from NGS and 3rd GS were aligned and similar sequence data from all libraries/samples were pooled. Due to the lack of a reference genome, the clean reads resulted in from the transcriptome sequences were aligned and assembled using Trinity software. In total, approximately 184,259,679 clean reads were obtained from the deep sequencing with 34–38 million reads per library for all samples and mapped reads ratios of 59.38, 61.08, 61.52, 57.73, and 57.24% at 0, 1, 6, 12, and 48 h after treatment, respectively (Table 1). The GC content ranged between 45–46%, Q20 ranged between 96–97% and the proportion of unknown nucleotides in clean reads (N %) was 0%. A total of 17,508 unigenes and 18,209 transcripts were obtained from all libraries with average lengths of 1,783 and 1785 base pairs (bp), respectively (Table 2). Furthermore, a total of 1827 (10.4%) unigenes and 1931 (10.6%) transcripts were longer than 3,000 bp and 84% of unigenes and transcripts were longer than 1,000 bp (Fig. 1A).

**Table 1:** Next generation sequencing statistical summary of sequenced and assembled results.

	0 h	1 h	6 h	12 h	48 h
Total reads	38,990,288	37,595,494	37,441,881	34,736,085	35,495,931
Mapped reads	23,178,490	22,970,724	23,021,704	20,039,976	20,308,985
Mapped reads (%)	59.38	61.08	61.52	57.73	57.24
Nt	11,697,086,540	11,278,648,200	11,232,564,300	10,420,825,700	10,648,779,500
GC (%)	46.60	46.51	46.73	45.53	45.59
Q20 (%)	96.01	96.76	96.60	97.03	96.81
Q30 (%)	92.01	92.00	91.52	92.46	92.07
N%	00.00	00.00	00.00	00.00	00.00

*Nt*, total number of clean nucleotides; *The GC%* is the proportion of guanidine and cytosine nucleotides among total nucleotides; *The Q20 and Q30%* is the proportion of nucleotides with a quality value > 20 and 30, respectively; *The N%* is the proportion of unknown nucleotides in clean reads.

**Table 2:** Third generation sequencing statistical summary of sequenced and assembled results.

	Unigenes	Transcripts
Total number of sequences	17,508	18,209
Total sequences length	31,222,209	32,513,465
Maximum length	8,455	8,455
Minimum length	227	227
Average length	1,783	1,785
GC (%)	43.72	43.74
N40	2,138	2,147
N50	1,853	1,857
N60	1,650	1,651
N70	1,454	1,455
N80	1,250	1,250
N90	1,052	1,052

*N50*, represents sorting the assembled transcripts from long to short by length, accumulating the length of the transcript to 50% of the total length, corresponding to the length of the transcript, and so on.

The species which provided the best BLASTX matches was *Nicotiana tomentosiformis* (19.86%), with more than 8,000 genes showing high homology, followed by *Nicotiana sylvestris* (19.83%), *Solanum tuberosum* (8.72%), *Sesamum indicum* (7.78%), *Solanum lycopersicum* (4.93%), and *Solanum pennellii* (4.51%). The reference sequence genome for Ipomoea batatas was ranked at the 8th position with only 3.45% sequence similarity. These data represent a sequence resource for genetic and genomic studies of sweet potato that will broaden the understanding of drought resistance mechanisms (Fig. 1B).

The assembled unigenes were validated and annotated using BLASTX search based on the sequence similarity search against the NCBI non-redundant (NR) protein database, Clusters of Orthologous Groups (COG) database, Gene Ontology (GO) database, Kyoto encyclopedia of genes and genomes (KEGG) database, Eukaryotic orthologous groups (KOG) database, protein family (Pfam) database and Swiss-Prot database. In total, 16,891 unigenes were detected which had good comparability with known gene sequences in at least one database, corresponding to 96.47% of the total unigenes. A total of 7,603 (45.01%), 13,860 (82.06%), 6,482 (38.38%), 10,740 (63.58%), 14,646 (86.71%), 14,620 (86.55%) and 16,866 (99.85%) unigenes were annotated in COG, GO, KEGG, KOG, Pfam, Swiss-Prot and Nr, respectively (Table 3).

**Table 3:** Statistics of unigenes annotated in public database.

Annotated database	Annotated number value (%)	300 ≤ length < 1,000 value (%)	Length ≥ 1,000 value (%)
COG	7,603 (45.01)	984 (05.83)	6,619 (39.19)
GO	13,860 (82.06)	2,040 (12.08)	11,817 (69.96)
KEGG	6,482 (38.38)	949 (05.62)	5,533 (32.76)
KOG	10,740 (63.58)	1,327 (07.86)	9,413 (55.73)
Pfam	14,646 (86.71)	2,009 (11.89)	12,637 (74.81)
Swiss-Prot	14,620 (86.55)	1,986 (11.76)	12,631 (74.78)
Nr	16,866 (99.85)	2,474 (14.65)	14,389 (85.19)
All	16,891	2,493	14,395

### Comparison of differentially expressed genes among four libraries between treated versus control samples

In order to quantify gene expression, the expression of each unigene was calculated using FPKM values (obtained fragments per kilo base of transcript per million mapped reads). Differentially expressed genes (DEGs) were identified by performing pairwise comparisons among the samples. Thresholds of false discovery rate (FDR) ≤ 0.05 and |log_2_ ratio|≥ 1 were set to identify significantly different expression between treated and untreated plants at each time point^32^.

**Figure 2:**
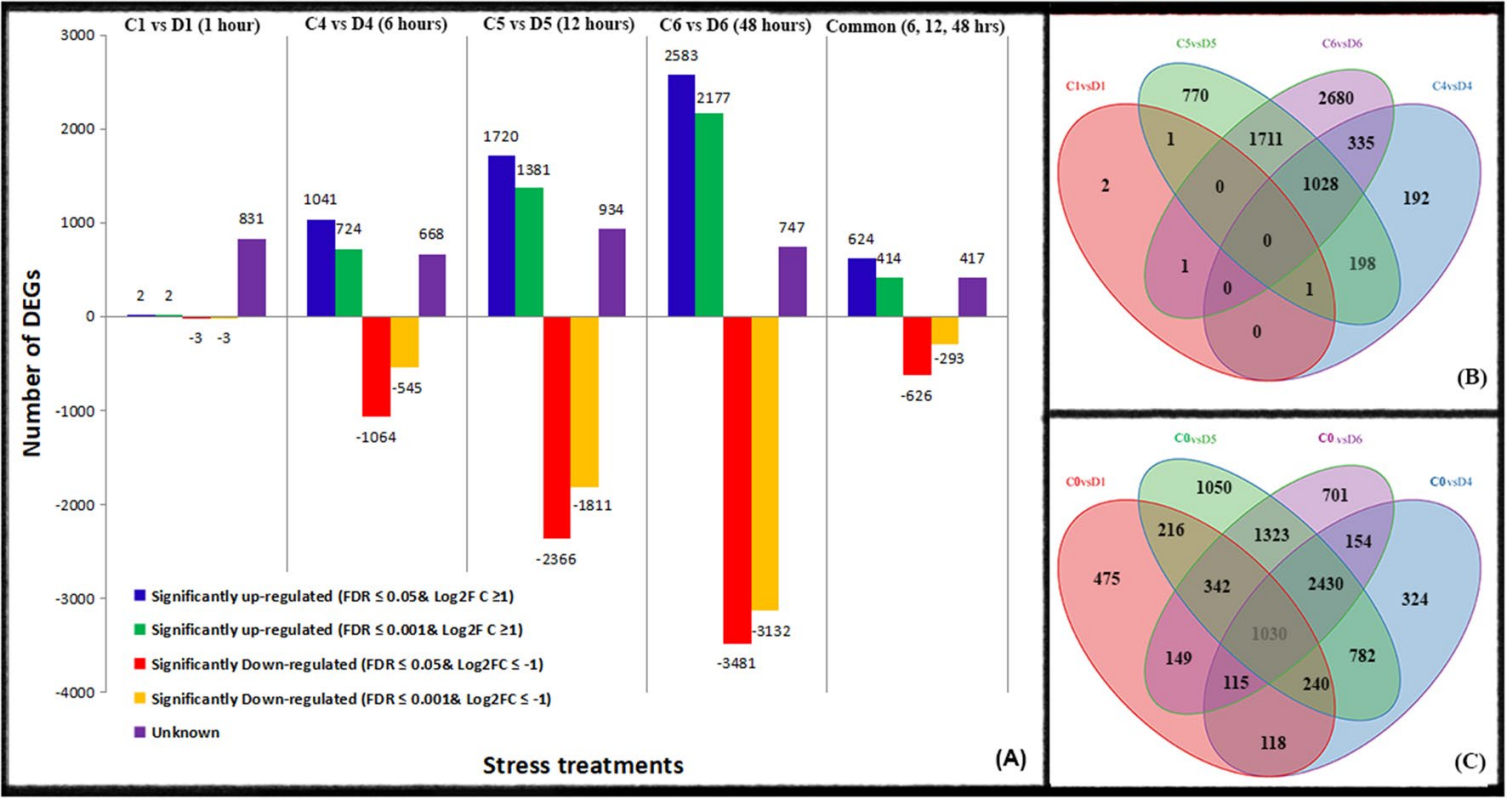
Statistical chart of differentially expressed genes (DEGs) transcriptome in response to drought stress. Number of DEGs (up- and down- regulated) of four libraries including 1, 6, 12 and 48 h of salt stress treatment as compared to control at two different levels of false discovery rate (FDR) including 0.001 and 0.05 (**A**). Venn diagram analysis of all induced unigenes comparing each treatment with the control at the same time point including C1 versus D1, C4 versus D4, C5 versus D5 and C6 versus D6 at 1, 6, 12 and 48 h of drought stress (**B**). Venn diagram analysis of all induced unigenes comparing all treatments with the zero time point one control against all treatments C0 versus D1, C0 versus D4, C0 versus D0 and C0 versus D6 at 1, 6, 12 and 48 h of drought stress (**C**).

A rigorous analysis of the algorithm was performed at FDR 0.01 and 0.05 to determine the differences between the two levels. At 1, 6, 12, and 48 h of stress exposure, compared to the no-treatment control, 2, 724, 1,381 and 2,177 significantly up-regulated unigenes and 3, 545, 1811 and 3,132 significantly down-regulated unigenes were detected, respectively at the transcriptional level, with values of FDR ≤ 0.001. Overall, 414 up-regulated unigenes and 293 down-regulated unigenes were common at all three time points (6, 12, and 48 h) (Fig. 2). At 0.05 false discovery rate (FDR), the number of 2, 1,041, 1,720 and 2,583 significantly up-regulated and 3, 1,064, 2,366 and 2,366 significantly down-regulated were observed at 1, 6, 12 and 48 h after exposure to drought stress, respectively. Furthermore, when exposed to drought for 6, 12 and 48 h, 624 unigenes were found to be up-regulated, while 626 unigenes were down-regulated at 0.05 FDR (Fig. 2A). Moreover, the comparison of FPKM values from the different drought treatment time-point libraries versus control samples for each time point was more accurate than comparison with control values at 0 time point. Ultimately, in the present study, a pairwise comparison algorithm of FPKM was used for comparison of control libraries at each sampling point (FDR at 0.05 and |Log_2_ FC ≥ 1|).

There were only 2 up-regulated unigenes TFs with significant expression levels. On the other hand, there were three significantly down-regulated unigenes, including thylakoid lumenal protein, Thermo-spermine synthase, and uncharacterized protein sequence after 1 h of drought stress. Upon exposure to drought stress for 6, 12 and 48 h, the number of commonly up-regulated unigenes was 624. Among these unigenes, 13 unigenes were annotated to an uncharacterized protein sequence. On the other hand, the total number of down-regulated unigenes was 626 at three different time points. Among 293 unigenes, 15 unigenes were annotated to an uncharacterized protein sequence (Fig. 2A, B).

After 6 h of drought stress, 1,041 up-regulated genes were differentially expressed in the leaf tissues. Among the up-regulated unigenes, ATP-dependent CLP protease was the most highly expressed gene (eightfold higher than control), followed by homeobox- leucine zipper protein (ATHB-7) and EID1-like F-box protein-3 which are expressed fivefold higher in PEG-treated samples than in control treatment and were. Furthermore, there were seven unigenes involved in drought tolerance, which were classified into different families, including GID1-like gibberellin receptor, LanC-like protein (GCL2), cinnamoyl-Co-A reductase-1, 2-hydroxy isoflavone dehydratase and carboxylesterase-1 (CXE-1). In addition, there were 10 unigenes involved in biological processes related to drought stress response, which belonged to four different families, including abscisic acid insensitive-5 like protein (bZIP-8), EID1-like F-box protein-3, 9-cis-epoxy carotenoid dehydrogenase, and cysteine protease inhibitor. On the other hand, there were 1,064 down-regulated unigenes in plants under PEG-induced drought stress compared to the control plants. The most pronounced change in gene expression occurred in down-regulated unigene was aligned to trypsin and protease inhibitor that exhibited a ninefold reduction in PEG-treated plants compared to control, followed by the unigene Methyl-CPG binding domain-11, MBD-11 which had a fivefold reduction in PEG-treated samples compared to control. In addition, two down-regulated unigenes were aligned to the defense mechanisms cluster, encoding p*rotein tyrosine* phosphatases and carboxylesterase-8. Furthermore, the *expression levels* of aspartic protease in guard cell and Jasmonate O-transferase were significantly down-regulated which contribute to processes involved in drought stress responses, including, respectively (Supplementary Table S1).

After 12 h of drought stress, there were 1,720 up-regulated unigenes and 2,366 down-regulated unigenes in PEG-treated plants compared to control plants. Among the significantly up-regulated unigenes, 1, 5 and 3 unigenes were expressed eightfold, sixfold, and fivefold higher in treated samples than in control, while the others were one–twofold higher than those of control. According to the information provided by the NR database, the highly expressed unigenes were annotated with the EID1-like F-box protein-3 and home-box leucine zipper protein HB-12 like. In addition, there were 17 unigenes associated with tolerance to drought stress, which encodes the gibberellin receptor (GID1-B), carboxylesterase, tetraketide alpha-pyrone reductase 1, lanC-like protein GCL2, 2-hydroxyisoflavanone dehydratase-like and 2-hydroxyisoflavanone dehydratase. Furthermore, 17 unigenes were involved in drought stress responses, which belonged to seven different families (*i.e*., abscisic acid insensitive like protein-5 (bZIP-8), heat shock protein-70 (Hsp-70), gibberellin receptor (GID1B), S-adenosyl-L-methionine-dependent methyltransferase, Xanthoxin dehydrogenase-like, cysteine protease inhibitor and EID1-like F-box protein-3). Out of 2,366 unigenes, 9 down-regulated were related to the category of defense mechanisms in which l-Galactono-1,4-lactone dehydrogenase, GID-1C, cinnamoyl Co-A reductase and carboxylesterase-8 play an important role (Supplementary Table S1).

The largest number of genes was recorded at 48 h of drought stress among all time points. Out of 2,583 up-regulated unigenes, 2, 9, 6, 16, 27, 38 and 76 unigenes showed 11, 10, 9, 8, 7, 6 and 5-fold higher expression, respectively in treated samples than in control. The superior unigenes with expression levels over tenfold higher than those of control belonged to various families including trypsin and protease inhibitor, FAD-dependent urate hydroxyl-like, YT-521-B like domain, protein phosphatase, arogenate dehydrogenase, heat shock protein-70, phosphor-enolpyrovate carboxykinase, STAY-GREEN protein, and EID1-like F-box protein-3. Moreover, 16 genes were assigned categories to defense mechanisms against drought stress, which were annotated to GID1-like gibberellin receptor, LanC like protein (GCL1), carboxylesterase, protein phosphatase, tetraketide alpha-pyrone reductase 1, phosphoglucan phosphatase LSF2 (chloroplastic), and 2-hydroxyisoflavanone dehydratase. In addition, 16 unigenes belonging to seven different families (WRKY, apolipoprotein D-like, ABA insensitive like protein-5 (bZIP-8), FAD binding domain, 9-cis-epoxycarotenoid dioxygenase-1, protein farnesyltransferase, and EID1 like f-box protein-3) were associated with the drought stress response. On the other hand, 3,481 unigenes were down-regulated at 48 h of stress exposure. Out of 3,132 unigenes, 16 unigenes were down-regulated to levels ninefold lower than those of control.

### GO and KOG classifications

**Figure 3:**
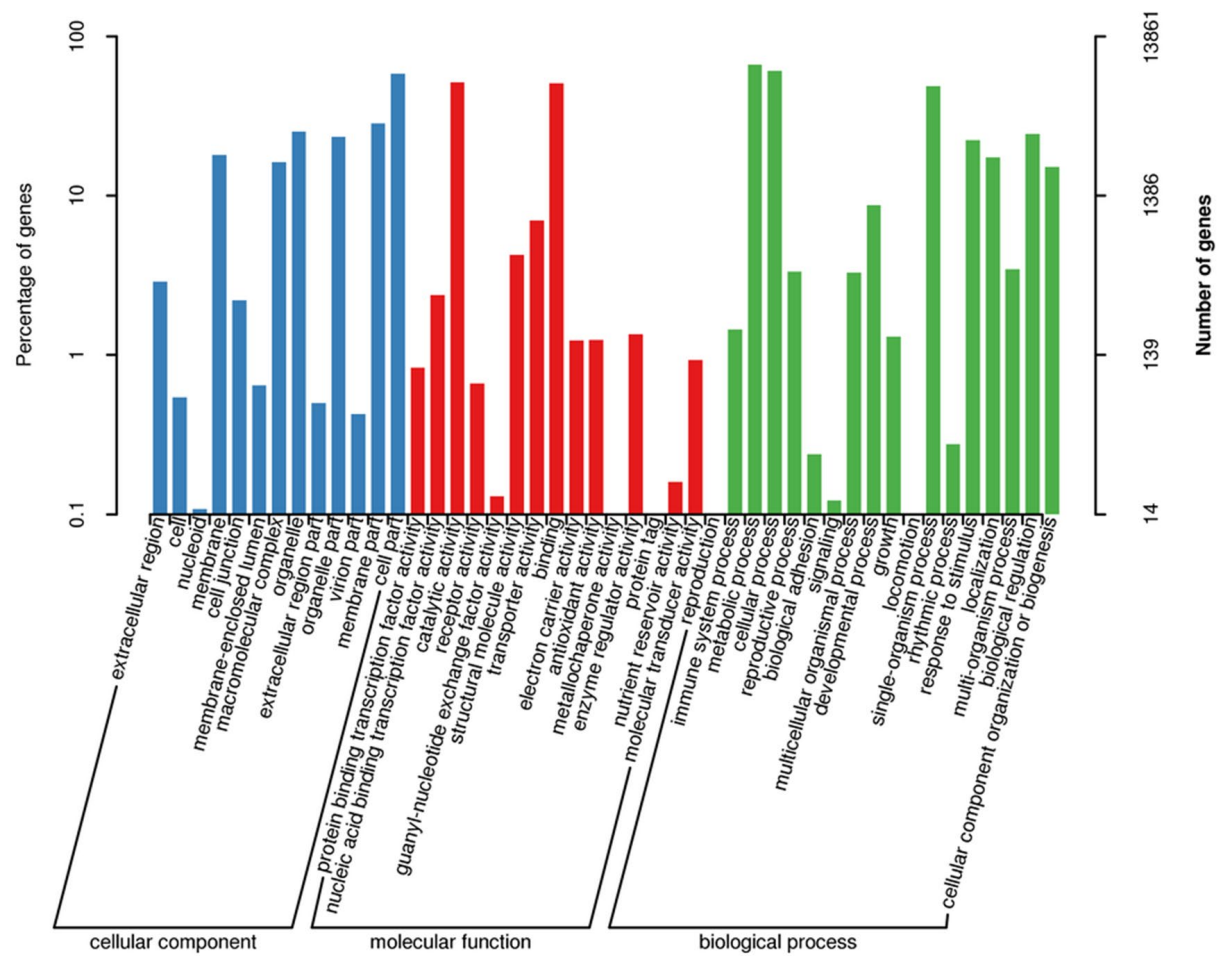
Gene ontology (GO) classifications in sweetpotato (Xuzi-8 cultivar), the percentage indicate the proportion of obtained unigenes with the GO annotations.

The *Gene Ontology* (*GO*) *classification* system was used to classify the possible functions of the obtained unigenes into 46 elements, which were assigned to three main categories, including biological process, molecular function, and cellular component (Fig. 3). Each unigene was at least assigned to one element of a category. In total, 38,524 unigenes were assigned to the category of biological processes which was further divided into 17 sub-categories. Among these sub-categories, “metabolic process”, “cellular process”, and “single organism process” included the largest numbers of 9,228, 8,452, and 6,777 unigenes, respectively. Furthermore, the sub-categories “biological regulation” and “response to stimulus” were ranked second with 3,390 and 3,083 unigenes, respectively. The “molecular function” category contained 16,956 unigenes, which were distributed in 16 different functional groups. The greatest number of unigenes (7,120 and 7,037) was classified into the sub-categories “catalytic activity” and “binding”, respectively. Moreover, 24,504 unigenes were assigned to the “cellular component” category and were further divided into 13 different sub-categories. For the “cellular component” category, the top largest categories were “cell part” (8,076 unigenes), “membrane part” (3,928), “organelle” (3,491), and “organelle part” (3,238). Additionally, a total of 286 unigenes were identified to be involved in drought stress tolerance response biological process according to Gene Ontology (GO) categories.

**Figure 4:**
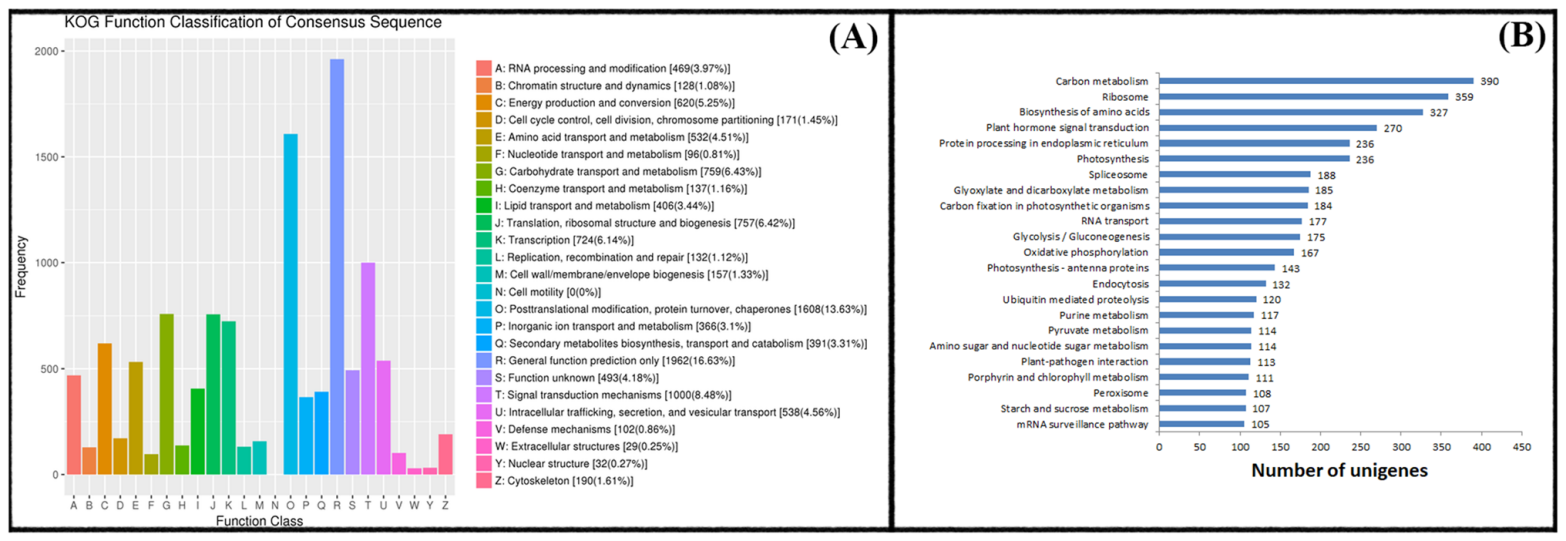
(**A**) Clusters of orthologous groups (COG) classification in Xuzi-8 sweetpotato cultivar for unigenes obtained under drought stress conditions. Genes from the same Orthologous have the same function, so that direct functional annotations to other members of the same KOG cluster. (**B**) the most enriched KEGG clusters in Xuzi-8 sweetpotato cultivar under drought stress conditions. The most enriched 22 clusters out of 123 clusters were presented in this figure.

The COG database was used to classify the potential functions of unigenes (Fig. 4A). In total, 10,740 unigenes (63.58%) were annotated and classified into 25 functional classifications. The results of the functional annotation analysis showed that the majority of unigenes (1608, 13.63%) were associated with “posttranslational modification”, “protein turnover”, and “chaperones” categories, followed by the “signal transduction mechanisms” category which contained 1,000 unigenes (8.85%). Furthermore, 102 unigenes were found to be involved in mechanisms of drought tolerance in sweet potato. In addition, 1962 (16.63%) and 493 (4.18%) unigenes with uncharacterized function or poorly characterized belonged to the “general function prediction only” and “function unknown” categories, respectively. On the other hand, no genes were found to be involved in “cell motility” category.

### KEGG pathway mapping

The KEGG pathway analysis showed that a total of 16,891 unigenes and 6,482 sequences were annotated and assigned to 123 pathways. At the first hour of PEG-induced drought stress only Ascorbate and Aldarate metabolism KEGG pathway (Ko00053) contained one up-regulated unigene encoded myo-Inositol oxygenase as compared to control. A number of 82, 104 and 117 pathways included differentially expressed genes as compared to control. The two largest pathway groups were metabolic pathways (ko01100, 1829 unigenes) and biosynthesis of secondary metabolites (ko01110, 943 unigenes). Moreover, the pathways such as “Carbon metabolism (ko01200)”, “Ribosome (ko03010)”, “Biosynthesis of amino acids (ko01230)”, “Plant hormone signal transduction (ko04075)”, “Photosynthesis (ko00195)” and “Protein processing in endoplasmic reticulum (ko04141)” contained 390, 359, 327, 270, 236, and 263 unigenes, respectively (Fig. 4B). The KEGG pathway enrichment analysis provides insights into the mechanisms involved in the drought tolerance of Zi-8 sweet potato cultivar.

### Differential expression of transcription factors (TFs) regulated by PEG treatment

**Figure 5:**
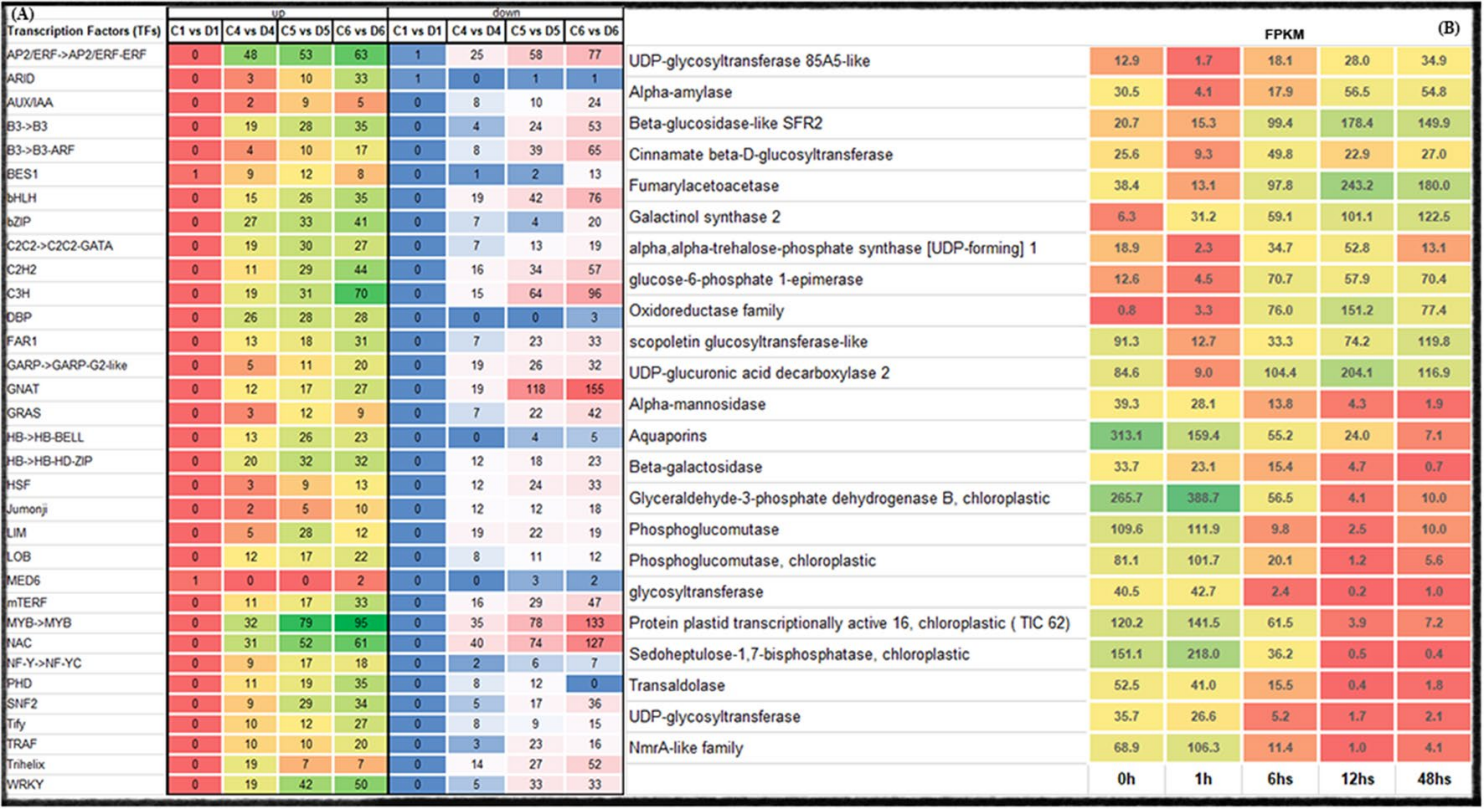
Transcription factors differentially expressed (**A**) and stress related protein genes (**B**) induced under drought stress in Xuzi-8 sweetpotato cultivar.

Among the 16,891 DEGs differentially regulated by PEG treatment, 5, 1754, 3,709 and 5,755 encode TFs at 1, 6, 12 and 48 h of PEG treatment, respectively, belonging to 60 different families. The most abundant transcription factor family found during all time points under drought stress was zinc finger proteins (ZFPs) (212 unigenes), followed by MYB (147 unigenes), ERF (88 unigenes), TSA (73 unigenes), bZIP (63 unigenes), WRKY (33 unigenes), NAC (30 unigenes), and bHLH (5 unigenes) (Fig. 5A, Supplementary File 1).

After 1 h of PEG treatment, only the beta-amylase 1(BRI1-EMS-SUPPRESSOR1 (BES1) TFs family) and inositol oxygenase 2-like (Mediator of RNA polymerase II Transcription, Subunit 6 (MED6) TFs family) genes were up-regulated. BES1TFs family plant-specific transcription factors that regulate BR-responsive genes. MED6 TFs family among its related pathways are regulation of lipid metabolism and plant growth.

At 6 h of PEG-induced drought stress, there were 1754 unigenes encoded as TFs including 920 up-regulated and 834 down-regulated. These up-regulated TFs were classified into 61 different families including AP2/ERF (48 unigenes) followed by NAC (31), bZIP (27), DBP (26), MYB (25), HB-HD-ZIP (20), WRKY (19), B3->B3 (19), C2C2->C2C2-GATA (19), C3H (19) and bHLH (15). Furthermore, the most pronounced TFs families at this time point included DBP (3), HB->HB-HD-ZIP (2), NF-Y->NF-YC (1) and SNF2 (4) giving more than 4 folds of expression level higher than control. Among all TFs the highest one was expressed eight fold higher than control was aligned to ATP-dependent Clp protease ATP-binding subunit CLPT1 (chloroplastic). On the other hand, among the 834 down-regulated TFs, the lowest expressed as compared to control were aligned to Nitrate reductase 2 (C3H), Germin-like protein (SET), 14 kDa proline-rich protein (Trihelix), glucan endo-1,3-beta-glucosidase 12 (AP2/ERF-ERF), Sporamin B and Methyl-CpG-binding domain-containing protein 11 (MBD) (Fig. 5A, Supplementary File 1).

After 12 h of PEG-induced drought stress there were 3,709 unigenes encoded TFs including 1578 up-regulated and 2,131 down-regulated. The up-regulated TFs were classified into 81 families including same families present at 6 h beside 20 TF families more. The highest number of genes was included under MYB (63), AP2/ERF-ERF (53), NAC (52), WRKY (42), bZIP (33), HB-HD-ZIP (32), C3H (31), C2C2-GATA (30) and C2H2 (29). The highest expressed TFs were annotated to C2C2-GATA (14), DBP (1), HB-BELL (1), HB-HD-ZIP (3), LOB (2), MADS-MIKC (1) and SNF2 (1) families. Among all, the highest TFs was aligned to Protein phosphatase 2C (DBP TF family) which was expressed nine folds higher than control. On the other hand, among the down-regulated TFs the lowest expression were given by gibberellin induced protein (mTERF), cytochrome P450 (Trihelix), glutamine synthase 2,Tubulin beta-2 chain (SWI/SNF-BAF60b), bHLH63-like (bHLH), chlorophyll a-b binding protein of LHCII type I-chloroplastic (GNAT), magnesium-chelatase subunit ChlH-chloroplastic (Jumonji) and 21 kDa protein-like isoform X2 (Fig. 5A and Supplementary File 1).

At 48 h, the plant gave the maximum response to drought stress giving 5,755 significantly expressed TFs including 2,418 up-regulated and 3,337 down-regulated. The number of active TFs families at 48 h was similar to 12 h of drought. However the highest number of genes were included under C3H (70), MYB (68), AP2/ERF-ERF (63), NAC (61), WRKY (50), C2H2 (44), bZIP (41), B3->B3 (35), bHLH (35), PHD (35), SNF2 (34), ARID (33), HB-HD-ZIP (32) and FAR1 (31). Among all TFs families the highest unigenes were belonging to B3->B3-ARF, C2C2-GATA, C3H, DBP, LIM, MADS-MIKC and SNF2 reaching more than 7 folds higher than control. On the other hand, among all down-regulated TFs the lowest TFs as compared to control were classified as B3-ARF, C2C2-CO-like, GNAT, HSF, LUG, SET and Trihelix TF families (Fig. 5A and Supplementary File 1).

### Protein kinases differentially expressed in response to drought stress

Out of 376 protein kinases (PKs) unigenes which were differentially expressed in leaf tissues under drought stress**,** 45 unigenes encoded receptor-like protein kinase (RLKs) in different libraries of control and drought stress (Table 4). Cysteine-rich receptor-like protein kinase and receptor like protein kinase HAIKU-2 genes were significantly up-regulated after 6, 12, and 48 h of drought stress. Besides these two genes, G-type lectin S-receptor-like protein kinase gene was up-regulated after 6 h and putative receptor-like protein kinase gene was up-regulated after 12 and 48 h of drought stress. On the other hand, the proline-rich receptor-like protein kinase (PERK-15) gene was down-regulated at all three time points (6, 12, and 48 h) of drought stress. Moreover, after 48 h of PEG treatment, the number of down-regulated RLKs unigenes was 10, which were then aligned to glycerophosphodiester phosphodiesterase protein kinase domain-containing (GDPDL2), carbohydrate-binding protein of the ER, leucine-rich repeat receptor-like protein kinase, receptor-like protein kinase 5, and receptor-like protein kinase HSL1.

**Table 4:** Classification of drought-inducible regulated proteins into different categories according to sequence similarity with previously known genes.

Regulatory protein category	No of total unigenes	C1 versus D1		C4 versus D4		C5 versus D5		C6 versus D6	
		Up-regulated	Down-regulated	Up-regulated	Down-regulated	Up-regulated	Down-regulated	Up-regulated	Down-regulated
**Protein kinases and phosphatases**									
Histidine Kinase	12	0	0	0	4	0	5	0	5
Calcium-dependent protein kinase	9	0	0	0	0	1	0	1	0
Mitogen-activated protein kinase	26	0	0	0	0	0	2	0	10
Mitogen-activated protein kinase kinase	15	0	0	0	0	0	4	1	2
Mitogen-activated protein kinase kinase kinase	6	0	0	0	0	2	0	0	0
Serine/threonine-protein kinase	239	0	0	18	8	40	17	32	37
**Protein phosphatase**									
SNF1-related protein kinase	14	0	0	0	0	0	0	1	0
LRR receptor-like kinase	28	0	0	1	1	2	2	3	8
Receptor-like kinase	112	0	0	9	4	15	9	19	25
Protein phosphatase	167	0	0	27	1	36	3	35	17
Serine/threonine-protein phosphatase	48	0	0	0	0	3	0	2	3
Phospholipase	30	0	0	3	4	7	3	11	6
Calmodulin	16	0	0	4	0	6	0	5	0
Calcineurin B-like proteins	12	0	0	3	0	1	2	0	2
CBL-interacting protein kinases (CIPK)	37	0	0	7	2	17	0	12	3
**Plant hormones**									
Abscisic acid	218	0	0	29	7	48	12	45	29
Aldehyde oxidase	5	0	0	0	0	0	0	3	0
Zeaxanthin epoxidase	2	0	0	0	0	0	0	0	1
Molybdenum cofactor sulfurase	3	0	0	0	0	0	0	0	1
9-Cis-epoxycarotenoid dioxygenase	7	0	0	5	0	6	0	6	0
Abscisic acid receptor	21	0	0	9	1	9	5	6	5
**Ethylene**									
1-Aminocyclopropane-1-carboxylate oxidase	11	0	0	0	0	1	0	0	5
1-Aminocyclopropane-1-carboxylate synthase	1	0	0	0	0	0	0	0	1
Ethylene receptor	2	0	0	0	0	0	0	0	0
Ethylene insensitive	31	0	0	1	0	3	0	2	0
Ethylene response factor	20	0	0	1	0	1	8	1	1
EIN3-binding F-box protein	13	0	0	2	0	2	0	1	7
**Jasmonic acid**									
Lipoxygenase (LOX)	13	0	0	0	0	1	0	1	4
Allene oxide synthase	1	0	0	0	0	0	0	1	0
Jasmonate O-methyltransferase	3	0	0	0	1	0	0	0	2
**Salicylic acid**									
Salicylic acid-binding protein	158	0	0	9	7	19	48	9	76
**Auxin**									
Tryptophan aminotransferase-related protein	2	0	0	0	0	0	1	0	2
Auxin transporter-like protein	4	0	0	0	1	0	3	2	1
Auxin efflux carrier component	1	0	0	0	0	0	0	0	1
Auxin response factor	5	0	0	0	1	0	1	0	1

The other PKs expressed under drought stress belonged to the SNF1-related protein kinase (11 unigenes, 1 up-regulated at 48 h), phosphatidylinositol 4-phosphate 5-kinase (8 unigenes, 1 up-regulated at 48 h), cyclin-dependent kinase (17 unigenes, 3 down-regulated at 48 h), CBL-interacting protein kinase 2-like (9 unigenes, 2 down-regulated at 6 h, 2 up-regulated at 12 h, 3 up- and 3 down-regulated at 48 h), histidine kinase (12 unigenes, 5 down-regulated at 6, 12, and 48 h) and mitogen-activated protein kinase kinase kinase YODA (47 unigenes, 6, and 12 down-regulated at 12, and 48 h) (Table 4).

### Stress-related protein genes induced by drought

#### Genes encoding redox regulation-related proteins

A set of 189 unigenes encoding redox-regulated proteins was identified under drought stress in leaf tissues of Xuzi-8 sweet potato cultivar. These genes encoded ascorbate peroxidase (APX), peroxidase (POD), oxidoreductase, monodehydroascorbate-reductase (MAD), glutathione S-transferase, Acyl Co-A reductase and Acyl Co-A syntheses^33^. All of these unigenes were commonly expressed in both PEG treated and untreated plants at 1 h after drought stress. The unigenes encoding ascorbate peroxidase and Acyl CO-A reductase were down-regulated after 6, 12, and 48 h of PEG treatment. The activities of bifunctional monodehydroascorbate reductase, carbonic anhydrase nectarin-3-like and monodehydroascorbate reductase were up-regulated after 12 and 48 h of PEG-induced drought stress. In addition, 2, 1, and 10 unigenes encoding Acyl Co-A syntheses (3-ketoacyl-CoA synthase 6, 3-ketoacyl-CoA synthase 1, 3-ketoacyl-CoA synthase 19, Long-chain acyl-CoA syntheses 8, 3-ketoacyl-CoA synthase 2 and Long-chain acyl-CoA syntheses 8) were up-regulated after 6, 12, and 48 h of drought stress, respectively, while the unigenes encoding 3-ketoacyl-CoA synthase 4 were down-regulated. Furthermore, 51 unigenes encoding glutathione S-transferase were down-regulated in leaf tissues under drought stress; however, 14 unigenes which encoded glutathione S-transferase zeta class like and T1-like were up-regulated (Fig. 5B and Supplementary File 1).

#### Genes involved in carbohydrate metabolism and osmotic adjustment

Thirty strongly up-regulated unigenes involved in carbohydrate metabolism and osmotic adjustment were constantly expressed in leaf tissues at 6, 12, and 48 h under drought stress conditions. These genes encoded beta-glucosidase-like SFR2, cinnamate beta-D-glucosyltransferase, UDP-glucuronic acid decarboxylase 2, 7-deoxyloganetin glucosyltransferase, fumarylacetoacetase, Alpha-amylase, glucose-6-phosphate 1-epimerase, Galactinol synthase 2, scopoletin glucosyltransferase, alpha, alpha-trehalose-phosphate synthase [UDP-forming], and histidine phosphatase superfamily. The expression level of the top up-regulated genes encoding galactinol synthase 2 and UDP-glycosyltransferase 85A5-like was sevenfold higher in treated than in control plants after 48 h of drought stress. On the other hand, 59 unigenes annotated as the “carbohydrate transport and metabolism” category were down-regulated at 6, 12, and 48 h of drought stress. These down-regulated unigenes encoded aquaporin (TIP1-1, TIP2-1, PIP2-7, TIP1-1, and NIP2-1), beta-galactosidase, glyceraldehyde-3-phosphate dehydrogenase B, (chloroplastic) phosphoglucomutase, chloroplastic, phosphoglycerate kinase (chloroplastic), protein plastid transcriptionally active 16 (chloroplastic), sedoheptulose-1, 7-bisphosphatase (chloroplastic), and UDP-D-apiose/UDP-D-xylose synthase 2. Moreover, the down-regulated unigenes with the lowest expression level (sevenfold lower in PEG treated than in control plant) encoded Sedoheptulose-1, 7-bisphosphatase, chloroplastic, and aquaporin PIP2-7 (Fig. 5B, Table 5 and Supplementary File 1).

**Table 5:** Classification of drought-inducible functional proteins into different categories according to sequence similarity with previously known genes.

Protein functions category	No of total unigenes	C1 versus D1		C4 versus D4		C5 versus D5		C6 versus D6	
		Up-regulated	Down-regulated	Up-regulated	Down-regulated	Up-regulated	Down-regulated	Up-regulated	Down-regulated
**DEGs involved in osmotic adjustment**									
Arginine decarboxylase	11	0	0	1	0	7	0	1	0
Choline monooxygenase	1	0	0	0	0	0	0	0	1
Pyrroline-5-carboxylate synthase	2	0	0	0	0	0	0	1	0
S-adenosylmethionine decarboxylase	17	0	0	0	1	5	0	0	2
Trehalose-phosphate phosphatase	1	0	0	0	0	1	0	0	0
Galactinol synthase	3	0	0	3	0	3	0	3	0
**DEGs involved in detoxification**									
Respiratory burst oxidase	1	0	0	0	0	0	0	0	1
superoxide dismutase	22	0	0	0	1	0	2	0	5
Catalase	48	0	0	0	1	1	6	14	8
Peroxidase	94	0	0	1	15	6	19	22	31
Glutathione reductase	3	0	0	0	0	2	0	2	0
Dehydroascorbate reductase	15	0	0	0	0	6	3	8	3
Glutathione peroxidase	7	0	0	0	0	2	0	1	0
Glutathione S-transferase	62	0	0	7	0	8	8	21	14
Ascorbate peroxidase	15	0	0	0	3	1	4	0	8
Metallothionein	11	0	0	2	0	0	0	4	0
**DEGs involved in osmotic protection, transporters and channels**									
Aquaporin	58	0	0	0	19	2	29	3	35
ABC transporter family protein	382	0	0	28	43	61	54	72	84
Multidrug resistance protein ABC-transporter protein	21	0	0	2	3	5	2	5	6
Chloride channel	9	0	0	0	0	3	1	0	1
Betaine aldehyde dehydrogenase	4	0	0	0	0	0	0	2	0
Delta-1-pyrroline-5-carboxylate synthetase	1	0	0	0	0	0	0	1	0
Proline oxidase/dehydrogenase	4	0	0	4	0	0	0	1	0
**LEA proteins**									
Late embryogenesis abundant protein	12	0	0	2	3	3	3	1	3
Dehydrin	18	0	0	14	0	16	0	15	0
**Others**									
Pathogenesis-related protein	7	0	0	0	0	5	0	7	0
Drought-related protein	12	0	0	1	0	1	0	0	0
Dehydration-related protein	2	0	0	0	0	0	0	2	2
Heat shock protein	60	0	0	2	0	18	2	8	0

### Validation of differential gene expression

**Figure 6:**
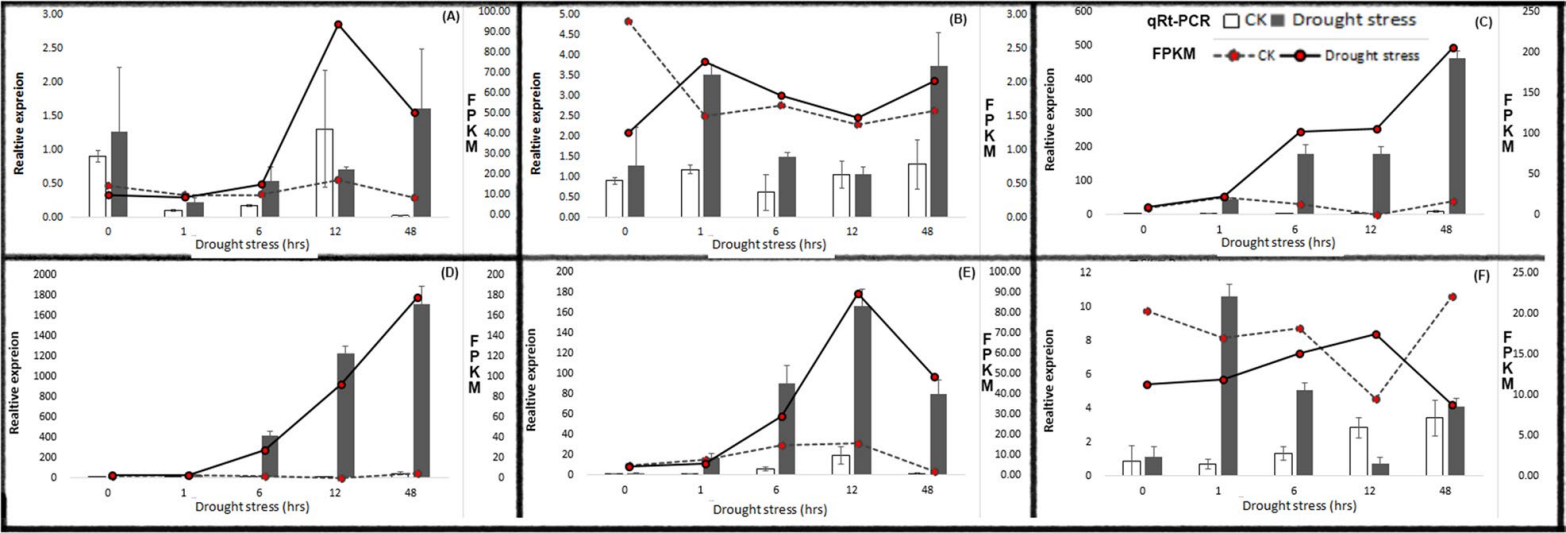
Quantitative real-time PCR analysis and fragments per kilo base million mapped reads (FPKM) of for 6 randomly chosen differentially expressed genes (DEGs). A, B, C, D, E and F; expression level determined by qPCR for the unigenes g14139, g2083, g16087, g2298, g8096 and g6453, respectively.

In order to validate differential gene expression results obtained by transcriptome sequencing, the expression levels of six randomly selected DEGs under PEG-induced drought stress were evaluated by qRT-PCR. Specific primer pairs for selected unigenes were designed as shown in Table 6. The expression levels determined by qPCR followed the same trend with the changes in the results of transcriptome sequencing, which suggests that our transcriptome sequencing data are highly reliable (Fig. 6).

**Table 6:** Forward and reverse primers used for QPCR validation experiment.

	Unigene code	Gene name	Forward (5′->3′)	Reverse (3′->5′)
1	g14139	Protein disulphide-isomerase (PDI)	TTTTCGTAATCCTGGCGGCG	ACCGTGTCGGAGAAGTTGGT
2	g2083	Sugar carrier protein (STC)	CCGCCCGAGAGGAAGTACAG	GGGCCAGACTCGTCGTACTC
3	g16087	Nucleoredoxin 2	GGAGGCGTTTGCTGCTTACC	TGCCCTCGACGTTGAACCTT
4	g2298	Thioredoxin-like	TTCGACGCCTACTTCGGGAC	GTCCGGCCCGAGAATTACCA
5	g8096	Thioredoxin superfamily protein isoform 1	TCTAGCAATGGTGCTGCGGA	AGCCCTACTGCTCCAACTACG
6	g6453	Zinc finger protein CONSTANS-LIKE 5 (COL5)	TATGGTGTCGGTACGGCGTC	TGACTCAGTTCTAGCGGCCC

## Discussion

Sweet potatoes are a rich source of nutrients. The production of sweet potato is limited by several abiotic stresses, resulting in tremendous yield losses^6^. Among these abiotic stresses, drought is a major threat to the sustainable production of sweet potatoes. Due to the complex nature of the sweet potato genome, extensive genomic, transcriptomic, and proteomic approaches are required to better understand its genome^34,35^. The lack of a reference genome is also a barrier to the accurate identification of candidate genes involved in drought tolerance. The present study was conducted to identify drought stress-responsive genes using next and third-generation sequencing techniques considering the severity of drought stress, the complex nature of the sweet potato genome, and the absence of a reference genome. Dual RNA-sequencing analysis provides molecular insights into defense mechanisms in plants against drought stress, which may further facilitate breeding for drought tolerant sweet potato cultivars^32^.

In the present study, HiSeq Illumina sequencing was used to characterize the transcriptome profiles of purple-fleshed sweet potato cultivar Xuzi-8 in response to different durations of drought stress. The second and third-generation sequencing methods produced a total of 184,259,679 clean reads, which were assembled into 17,508 unigenes with an average length of 1,783 base pairs (bp). According to GO, the top cellular component involving the largest number of genes was “cell part” sub-category. The balance between the metabolic and catabolic activities estimates the degree of resistance of a plant to drought stress at each time point^36^. Our results are in agreement with those of a previous study, reporting that the top three GO terms were metabolic process, cellular process, and catalytic activity in Ipomoea trifida under drought stress^10^.

The results of COG function showed that the greatest numbers of genes were assigned to “posttranslational modification”, “protein turnover”, “chaperones” and “signal transduction mechanisms” categories^37^. When plants are exposed to drought stress, the signal transduction mechanisms, which are responsible for detecting a physical or chemical signal transmitted through the cell as a series of molecular events, become activated, which ultimately results in a cellular response^38,39^. After the detection of signals, the plant modulates the activities by enzymatic modification of proteins following protein biosynthesis and protein turnover through achieving the balance between protein synthesis and degradation. In addition, chaperone proteins, which assist in protein folding as a chemical reaction under stress conditions, act to prevent or correct damage caused by miss folding^40^.

Drought inducible genes have recently been classified into two categories, namely, functional and regulatory proteins. Functional proteins are involved in abiotic stress tolerance, i.e., LEA, chaperons and dehydrin, detoxification, osmolytes biosynthetic (proline and sugar), protease, and water channel transporters proteins. Regulatory proteins are involved in regulating other genes that participate in drought tolerance, including TFs (bZIP, DREB2, MYC, AREB, MYB, and NAC), PKs, phosphatase, lipid metabolism, and ABA biosynthetic involved genes^40^.

### Differentially expressed genes among the four PEG treated libraries as compared to control library

In order to establish an accurate base for DEGs comparison, different significant levels were used (0.05 and 0.01). Furthermore, the use of the same control library for comparing with the treated libraries did not give representative results. Therefore, finally, a pairwise comparison algorithm of FPKM was used for comparison of control libraries of each sampling point comparing with the treated libraries were chosen at *P* value 0.05.

In the current study, at the first hour of PEG-induced drought stress, there were only three down-regulated unigenes in the leaf tissues which were aligned to thylakoid luminal protein and thermosporamine synthesis proteins. These genes were found to be involved in cell tip growth, meristem initiation, elongation, and cell wall organization during the first hour of drought stress. This explains that in sweet potato at the first hour of drought stress, the plant starts hormonal signaling, and growth diminishes^41,42^.

A set of gene families, including GID1-like gibberellin receptor (GID1), 2-hydroxy iso-flavanone dehydratase (HFD), Carboxylesterase-1 (CXE-1), Cinnamoyl-Co-A reductase-1 (CCo-A-R), and LanC-like protein (GCL2), were continued to be upregulated at high levels in Xuzi-8 leaf tissues at the different time points of PEG-induced drought stress (6, 12, 48 h). These genes play a vital role in plant defense mechanisms against drought stress^43^. Besides their role in drought tolerance, the four genes, including GID1, HFD, CCo-A-R, and CXE-1, control the hydrolase activity, which is related to energy metabolism and storage. Moreover, hydrolase activity is a chemical process in which a molecule of water is added to a substance that decreases the water loss from plant^44^. Thus, according to the current results of Xuzi-8, the plant resorted to reducing water loss through activating the genes of hydrolases activity in leaf tissues. LanC-like protein (GCL2) was reported previously to be one of the main drought-tolerant genes in Arabidopsis^45^ wheat^46^, and peach^47^, which explains the current results.

According to the current results, at 6 h of PEG- induced drought stress, leaf tissues start to sense the stress through the early perception of water deficiency leading to activation of the stress-responsive genes and stimulation of stomatal closure to reduce water loss^48^. The highest expressed gene in leaves at 6 h of drought stress was ATP-dependent Clp proteases (eightfolds higher than control). This gene minimizes the protein damage and maintains the quality of cellular proteins, thereby increases the tolerance for drought stress^49,50^. The current results indicated that the EID1-like protein 3 was expressed five-fold higher in leaf tissues than that control and continued with a high level at 6, 12, and 48 h of PEG-induced drought stress. This gene is involved in regulating the responses controlled by plant hormones, which not only regulate many aspects of plant development but also integrate responses toward drought stress^51^. On the other hand, the current results found that several genes were significantly down-regulated as compared to control. Trypsin-protease inhibitor showed a nine-fold reduction in Xuzi-8 leaf tissues as compared to control, which is in agreement with a previous study conducted on tomato drought resistant cultivar^52^. Protein aspartic protease down-regulated targets in ABA-signaling increasing drought avoidance through guard cells closure^53^.

In the current study, the highest number of drought stress-related genes was active at 12 h of PEG-drought stress. This means that Xuzi-8 sweet potato cultivar is at the peak of restoring to resist the stress after 12 h of its onset. In total, two gene families were acting as the superior genes at 12 h of drought stress giving an expression level that was more than eight folds than that in the control. These sets of genes were annotated to EID1-like F-box protein-3, which continued at the highest level from 6 h till 48 h of exposure to PEG-drought stress.

After 48 h, the expression of the fast action and non-specific drought stress genes fall back to the normal level, and only the drought specific genes were active. Remarkably, trypsin and protease inhibitor, FAD-dependent urate hydroxyl-like, YT-521-B like domain, protein phosphatase, arogenate dehydrogenase, heat shock protein-70, phosphor-enolpyruvate carboxykinase, STAY-GREEN protein, and EID1-like F-box protein-3 genes were expressed at ten-fold higher level than control at 48 h of PEG-induced drought stress. Trypsin and protease inhibitors were expressed at tenfold higher level than the control at 48 h of PEG-induced drought stress. However, they were expressed at nine-fold lower level than that of the control at 12 h. Trypsin protease inhibitor potentially increases drought stress tolerance and inhibits pathogen infection; therefore, it can be used as an excellent candidate as a lead compound for the development of a drought-tolerant character^54^. FAD-dependent urate hydroxyl-like was found to be involved in the catalysis of purine, which plays a role in the recycle of nitrogen for remobilization to support new growth and reproduction^55^. YT-521-B like domain is involved in cell growth, differentiation, and performs an important function in mediating a wide range of cellular responses under multiple abiotic stresses^56^. Arogenate dehydrogenase is responsible for root elongation at low water potential^57^. Heat shock protein-70 regulates the abscisic acid-induced antioxidant response under drought and heat stress^58^. Phospho-enolpyruvate carboxykinase was found to be involved in acclimation of the plant to drought stress^59^. STAY-GREEN protein encodes the regulatory proteins trigging chlorophyll catabolism and known to delay senescence, and improve plant survival and yield^60^.

### Transcription factors (TFs) regulated by PEG treatment

In the current study, at the first hour of PEG-induced drought stress, only two TFs were up-regulated, including beta amylase-1, which is involved in the starch catabolic process and inositol oxygenase-2 like, which is involved in inositol catabolic process. These two TFs belong to BES1 and MED6 TF families. BES1 TFs family is a class of plant-specific transcription factors that cooperate with other transcription factors to regulate BR-induced genes. Moreover, it starts stress tolerance through the control of many basic cellular processes and stress responses, promotes vigor in plants, and prepares them to mount a dynamic response upon environmental challenges. MED6 TFs family among its related pathways is involved in the regulation of lipid metabolism and plant growth. The transcriptional changes in genes related to lipid metabolism indicate its function at the interface in response to drought stress^61^. These two TFs, according to the current results, can be considered as early response genes in sweet potato leaf tissues for stress and may not be specific for drought stress. They were also found to be involved in the primary sensing of abiotic stress and in triggering and regulating cellular hormonal signaling^62^.

The majority of drought-related unigenes in this study were classified under different families of transcription factors (TFs) such as AP2/ERF, NAC, bZIP, DBP, MYB, HB-HD-ZIP, WRK, B3->B3, C2C2->C2C2-GATA, C3H, and bHLH, which were identified at all studied time points except for the first time point of drought stress. It was reported that TFs are considered as early activated genes that are targeted by phosphatases and PKs^63^. Among the active TFs, WRKY are largely up-regulated and known to be involved in drought stress defense pathways in plants^64–66^, and the members of AP2-EREBP TFs family are also shown to be highly up-regulated. They play an important role in drought stress signaling pathways induced by hormones^67^. In addition, NAC factors were differentially expressed and played specific roles in response to drought stress^68^.

The bHLH transcription factors (TFs) play a crucial role in controlling gene expression by regulating diverse biological processes such as growth, development, and drought stress response^69,70^. In the current study, bZIP TFs were significantly up-regulated at all time points (6, 12, and 48 h of stress) and played crucial roles in response to drought stress and regulated numerous growth and developmental processes^71^. These bZIP TFs included common regulatory factor (CPRF1), ABSCISIC ACID-INSENSITIVE 5-like protein 5, ABF2, bZIP28, and the transcription activator (TAF1). Interestingly, GO-annotation results showed that the aligned sequences of ABF2 are involved in response to water deprivation^72^. C2H2-type zinc finger proteins belong to the major family of transcription factors that can be classified as osmotic stress-responsive genes^73^ and bind to DNA, RNA, or protein^74^. C3H transcription factor (TF) family included 8, 16, and 21 unigenes that were significantly up-regulated and assigned to “posttranslational modification”, “protein turn over” and “chaperones” categories. These genes included C3HC4-type Ring-like zinc finger protein, XERICO^74^, and SPAP32A, which have only been characterized in *Schizosaccharomyces pombe* species but till now have not been studied in sweet potato. MYB transcription factor family in the present study included two up-regulated genes *MYB44* (*MYB44*) and MYB1-R1, which are involved in posttranslational modification, protein turn over, and chaperone-like activities^75,76^.

During the exposure to drought stress, the plant uses ABA as one of the key signaling molecules. In the current study, 9-cis-epoxycarotenoid dioxygenase was differentially expressed, which could be considered a key step in ABA synthesis^77^. Further, many DEGs encoded protein phosphatase PP2C were differentially expressed and played an important role in ABA regulation^78^. These results are in agreement with previous studies focused on drought stress^79^. Ethylene responsive factors (ERFs) play a regulatory role in water logging responses, are also considered in ethylene sensor promotion, and reported to play a key role in cell senescence^10,80^. They were down-regulated and contributed to the mitigation of drought stress in sweet potato leaves. In addition, the upregulated TF belongs to APETALA2/ethylene-responsive factor (AP2/ERF), which was observed to reduce transpiration and prevent a rapid decline in photosynthesis^18,81^.

### Protein kinases differentially expressed in response to drought stress

Remarkably, the DEGs encoded kinases, such as receptor-like kinases and LRR receptor-like kinase, were found to be largely up-regulated after 6, 12, and 48 h of PEG-induced drought stress. It is well established that LRR-RLKs play a valuable role in response to drought stress^82^. On the other hand, a substantial number of DEGs were downregulated under drought stress as compared to the control at different time points. In the current study, another major class of differentially expressed regulatory proteins is PKs, which fincluded SNF1-related protein kinase, phosphatidylinositol 4-phosphate 5-kinase, cyclin-dependent kinase, CBL-interacting protein kinase 2-like, histidine kinase and mitogen-activated protein kinase kinase kinase YODA. PKs are considered drought stress sensor response genes that activate phosphorylation, which play important roles in response to drought stress^62,83^.

### Stress-related protein genes induced by drought stress in Xuzi-8 sweet potato cultivar

In the current study, the second class are function proteins including osmolytes biosynthesis, water channel, sugar and proline transporters, detoxification enzymes, chaperones, and late embryogenesis abundant (LEA) proteins. This class of protein helps plants in controlling water efflux influx through various mechanisms during drought stress. Thus they protect the integrity of the cell membrane and provide an aqueous environment for plant organelles^84,85^. In this study, many genes were identified to be involved in the metabolism of these osmolytes. Osmotically active solutes (osmolytes) are induced for the maintenance of cellular functions through biosynthesis of different substances as well as enzyme activity and proteins^86^. In the current study, proline, trehalose, and glycine betaine were found to have a relation in combating drought stress. Another important osmolyte is proline, which accumulates under the first phase of stress. Proline is an essential component of drought tolerance, especially at the first phase. Its contribution is diverse as it participates in osmotic adjustment and stabilization of subcellular structure^87^. In this study, two major proline components, including proline oxidase/dehydrogenase and Delta-1-pyrroline-5-carboxylate synthetase were found to be up-regulated at 6 h then decreased and upregulated again at 48 h of drought stress. It is required for maintaining growth at low water availability especially at the first phase of stress and to maintain growth at low water availability.

In the current study, it was found that a large number of DEGs encode proteins required for detoxification, such as protein detoxification-30, ascorbate peroxidase, glutathione peroxidase, glutathione S-transferase, ABC-2 transporter (Pleiotropic drug resistance, PDR), and superoxide dismutase. These genes have a major role in detoxification of ROS in the plant cell, which is extremely harmful as it causes lipid oxidation, DNA damage, and apoptosis. In agreement with our study, the role of protein detoxification has been documented in many plant species, i.e., *Ipomoea batatas*^18,88^, Ipomoea pes-caprae^89^*, Arabidopsis thaliana*^90^*, Nicotiana tabacum*^91^, *Oryza sativa* L.^92^ and *Solanum lycopersicum*^93^*.* In addition, TSA genes are involved in the detoxification of reactive oxygen species (ROS), reactive sulfur species (RSS), and reactive nitrogen species (RNS). TSA genes were significantly up-regulated, as they have a role in drought avoidance in plant^94^.

Aquaporin encoded DEGs were identified in our study, including PIP2-1, SIP2-1 at 6, 12, 48 h of drought stress, which considered as a part of stress protecting protein families in sweet potato that can facilitate water uptake. Late embryogenesis proteins (LEAs), including late embryogenesis abundant protein and dehydrin, were highly up-regulated in the current experiment at 6, 12, and 48 h of drought stress. These LEAs proteins play a protective role under osmotic stress conditions, homeostasis of proteins and nucleic acids, stabilization of cell membranes, and maintenance of redox balance^95^. In addition, a set of gene families including ABC transporter B, C, G family; LanC- like protein Gcl2 and MATE efflux family protein-5 play a vital role in a plant’s defense mechanisms against drought stress.

Taken together, Xuzi-8 sweet potato plants start sensing drought stress after 1 h but the highest action taken against drought stress started after 6 h till and reached the maximum response at 48 h. There is a huge network of genes involved in drought stress tolerance mechanisms, which contribute in determining the level of tolerance of each cultivar. A substantial proportion of DEGs was identified in our study, which did not match with any database. Many of these DEGs should show a significant change in their expression levels as compared to control during the different time points of drought stress. Therefore, these DEGs are an unexplored resource that might be involved in responses to drought stress or may be enriched during drought tolerance in sweet potato. Thus our study of Xuzi-8 sweet potato transcriptome sequencing could be used for understanding drought tolerance mechanisms in sweet potato and further exemplify the function aspects of the sweet potato genome.

## Supplementary information

Supplementary file1

Supplementary file2
