# Supplementary material for: RNA-sequencing analysis revealed genes associated drought stress responses of different durations in hexaploid sweet potato
Source: Sci Rep. 2020 Jul 28;10:12573. doi: 10.1038/s41598-020-69232-3 (PMC7387466; doi:10.1038/s41598-020-69232-3)
Supplement: Supplementary file 1 — Supplementary file1 [file 41598_2020_69232_MOESM1_ESM.docx]

**RNA-sequencing analysis revealed genes associated drought stress responses of different durations in hexaploid sweet potato**

Mohamed Hamed Arisha^1, 2,*^, Muhammad Qadir Ahmad^1, 3^, Tang Wei^1^, Liu Yaju^1^, Yan Hui^1^, Kou Meng^1^, Wang Xin^1^, Zhang Yungang^1^, Li Qiang^1,^ ^*^

*^1^ Xuzhou Institute of Agricultural Sciences in Jiangsu Xuhuai District / Key Laboratory of Biology and Genetic Improvement of Sweetpotato, Ministry of Agriculture / Sweetpotato Research Institute, CAAS, Xuzhou 221131, Jiangsu, China*

*^2^ Department of Horticulture, Faculty of Agriculture, Zagazig University, Sharkia 44511, Egypt*

*^3^ Department of Plant Breeding and Genetics, Bahauddin Zakariya University, Multan 60000, Pakistan*

*Corresponding author E-mail: [mohhamedarisha@gmail.com](mailto:mohhamedarisha@gmail.com), instrong@163.com

**Supplementary Table S1:** Defense mechanisms involved genes that were significantly expressed under different durations of drought stress (30% PEG-6000)

| Library | Unigene ID | Fold change | Nr. annotation | Unigene ID | Fold change | Nr. annotation |
| --- | --- | --- | --- | --- | --- | --- |
| Up-regulated | | | | Down-regulated | | |
| C1 vs D1 | - | - | - |  |  |  |
| Common in  the 3 libraries (D4, D5 and D60 | g11965  g12031  g14082  g480  g5326  g7573 | 1.6  1.9  1.4  1.2  1.8  1.6 | ABC transporter G family member 22-like  ABC transporter G family member 22-like  ABC transporter G family member 43  Pleiotropic drug resistance protein 1  ABC transporter B family member 19  ABC transporter G family member 22 | g2923  g9145 |  | ABC transporter G family member 32-like  unnamed protein product (uncharacterized) |
| C4 vs D4 | g12144  g16739  g4098 | 1.1  1.3  1.2 | pleiotropic drug resistance protein 1  LanC-like protein GCL2  Pleiotropic drug resistance protein 1 | g17399  g2811  g460  g6943  g7234  g869 | 1.6  1.5  2.4  1.7  1.2 | ABC transporter B family member 13  ABC transporter G family member 32-like  ABC transporter B family member 13  ABC transporter B family member 2-like  ABC transporter B family member 13-like  ABC transporter G family member 22-like |
| C5 vs D5 | g10245  g11314  g11939  g11948  g1212  g12144  g12706  g14388  g14675  g1468  g17032  g1730  g1851  g1919  g3031  g4098  g4149  g4748  g5014  g5385  g554  g6852  g7203 | 1.8  2.1  4.6  1.3  1.9  1.4  1.1  4.1  1.3  5.8  1.8  1.1  1.9  1.7  1.1  1.4  1.9  1.9  2.3  1.6  2.3  2.4  1.4 | ABC transporter G family member 36-like  ABC transporter G family member 22  ABC transporter B family member 26  ABC transporter G family member 7  ABC transporter C family member 14-like  pleiotropic drug resistance protein 1  ABC transporter G family member 7  protein DETOXIFICATION 24  protein DETOXIFICATION 14-like  ABC transporter C family member 5  ABC transporter C family member 14-like  ABC transporter C family member 10-like  ABC transporter C family member 14-like  ABC transporter C family member 14-like  PDR-type ACB transporter  pleiotropic drug resistance protein 1  ABC transporter C family member 14-like  ABC transporter G family member 35-like  ABC transporter B family member 19  ABC transporter C family member 14-like  ABC transporter C family member 14-like  ABC transporter C family member 14-like  ABC transporter C family member 14-like | g108  g1183  g12126  g1522  g15326  g15403  g15868  g1713  g2115  g2215  g2811 | 1.5  1.8  2.6  1.4  2.3  1.2  1.9  1.4  2.5  2.7  2.9 | ABC transporter B family member 15-like  ABC transporter C family member 12-like  ABC transporter G family member 36-like  ABC transporter C family member 2-like  MATE efflux family protein 5-like  MATE efflux family protein 5-like  ABC transporter B family member 15-like  ABC transporter C family member 2-like  ABC transporter G family member 36-like  ABC transporter G family member 36-like  ABC transporter G family member 32-like |
| D6 vs D6 | g11314  g11734  g11887  g11948  g14388  g14396  g1730  g1828  g1851  g4149  g5014  g554  g7203  g889 | 2.8  2.1  1.3  1.2  1.6  2.1  1.1  1.6  1.2  1.9  4.2  1.9  2.4  2.1 | ABC transporter G family member 22  ABC transporter G family member 11  pleiotropic drug resistance protein 2-like  ABC transporter G family member 7  protein DETOXIFICATION 24  Protein DETOXIFICATION 16  ABC transporter C family member 10-like  pleiotropic drug resistance protein 2-like  ABC transporter C family member 14-like  ABC transporter C family member 14-like  ABC transporter B family member 19  ABC transporter C family member 14-like  ABC transporter C family member 14-like  ABC transporter G family member 11 | g1183  g11872  g13010  g13461  g1378  g1522  g15326  g15403  g1713  g17399  g1956  g2215  g2412  g460  g7234  g7864  g869  g9930 | 1.3  3.6  2.6  1.3  1.4  3.1  2.2  1.4  2.8  1.9  1.9  2.3  1.4  1.7  2.2  1.9  1.6  3.1 | ABC transporter C family member 12-like  ABC transporter C family member 3  ABC transporter B family member 25-like  Protein DETOXIFICATION 40  MATE efflux family protein 4  ABC transporter C family member 2-like  MATE efflux family protein 5  MATE efflux family protein 5-like  ABC transporter C family member 2-like  ABC transporter B family member 13  putative ABC transporter C family member 15  ABC transporter G family member 36-like  MATE efflux family protein 4  ABC transporter B family member 13  ABC transporter B family member 13-like  ABC transporter B family member 13  ABC transporter G family member 22  ABC transporter C family member 15 |
